# The protective effect of *Zataria multiflora* Boiss. hydroalcoholic extract on TNF-α production, oxidative stress, and insulin level in streptozotocin-induced diabetic rats

**Published:** 2019

**Authors:** Marzieh Mahmoodi, Farhad Koohpeyma, Forough Saki, Amir Maleksabet, Mohammad Ali zare

**Affiliations:** 1 *Student Research Committee, School of Nutrition and Food Sciences, Shiraz University of Medical Sciences, Shiraz, Iran*; 2 *Department of Endocrinology, Endocrinology and Metabolism Research Center, Shiraz University of Medical Sciences, Shiraz, Iran*; 3 *Shiraz Endocrinology and Metabolism Research Center, Shiraz University of Medical Sciences, Shiraz, Iran*; 4 *Department of Medical Biotechnology, Faculty of Advanced Medical Sciences and Technology, Shiraz University of Medical Sciences, Shiraz, Iran*

**Keywords:** Zataria multiflora, TNF-α, Insulin, Liver, Diabetes, STZ

## Abstract

**Objective:**

Oxidative stress leads to reactive oxygen species (ROS) overproduction, which causes tissue injury in diabetic patients. The aim of this study was to evaluate the effects of *Zataria multiflora* extract on TNF-α, oxidative stress products, and insulin levels as well as lipid profile in diabetic rats.

**Materials and Methods:**

Rats were randomly divided into 6 groups of 10 animals. Diabetes was induced by a single injection of streptozotocin (STZ). Control and diabetic control rats orally received 1 mL/day of normal saline, whereas the other three groups received 250, 500 and 1000 mg/kg/day of *Z. multiflora* extract, and one non-diabetic group orally received 1000 mg/kg/day *Z. multiflora* extract, for 28 days. At the end of the treatment course, rats were anesthetized and their serum samples were analyzed for TNF-α, malondialdehyde (MDA), super oxide dismutase (SOD), total antioxidant capacity (TAC), lipid profile, total plasma protein, blood glucose, insulin, and liver enzymes levels.

**Results:**

Our results showed that cholesterol, LDL, TG, MDA and TNF-α levels decreased, but HDL, SOD, TAC, and total protein increased significantly in the diabetic group receiving 1000 mg/kg* Z. multiflora* compared to the diabetic control group (P<0.05). Moreover, blood glucose level was significantly reduced following administration of different concentrations of Z.* multiflora*. Liver sections of diabetic rats treated with Z. *multiflora* 1000 mg/kg showed normal hepatocytes and restoration of liver architecture.

**Conclusion:**

*Z. multiflora* extract ameliorated oxidative stress, TNF-α serum level, lipid abnormality, blood glucose, and liver damage in rats with diabetes mellitus.

## Introduction

Diabetes mellitus (DM) is a metabolic disease in which the secretion of insulin by pancreatic beta cells, is impaired or the sensitivity of tissues to insulin is reduced. Hence, insulin ability to lower blood glucose level is compromised, which consequently leads to hyperglycemia (Kahn, 2003 ▶; Hosseini et al., 2014 ▶). The prevalence of DM in Iran is on the rise, and it results in increased disabilities and lower quality of life. In 2006, more than 3.5 million people were diagnosed with DM, and it is believed that about half of them were unaware of their illness (Ghafarzadega et al., 2013 ▶). DM can cause various damages to body organs, and the level of destruction is associated with the duration and severity of the disease (Akan et al., 2014 ▶).

Superoxide (O2^•–^) and hydroxyl (OH) are known as reactive oxygen species (ROS) that are naturally involved in intracellular signaling. However, in conditions such as diabetes, hyperglycemia induces ROS overproduction leading to elevated levels of cellular ROS. High blood glucose induces nicotinamide adenine dinucleotide phosphate oxidase (NADPH) production which in turn, leads to the activation of cytochrome P450-like activity, the main activator of ROS production (Matough et al., 2012 ▶). Also, high blood glucose leads to overproduction of glycated proteins, causing tissue injury and pathological changes, which exacerbate ROS production (Patche et al., 2017 ▶).

The liver is an important organ which plays a critical role in oxidation and detoxification of waste products. Its function could be impaired in diabetic patients by excessive ROS production (Patche et al., 2017 ▶). An animal study showed that diabetes could induce liver oxidative stress and inflammation; hence, insulin therapy by itself was not able to reduce the resultant adverse effects (Ois Moreau et al., 2015 ▶). Thus, applying antioxidant therapy in conjunction with insulin therapy might be regarded as a more effective modality in preventing diabetic complications (Ois Moreau et al., 2015 ▶). Another animal study showed that the use of antioxidant can lead to amelioration of the oxidative stress status under diabetic conditions by increasing the antioxidant enzymes levels (Aboonabi et al., 2014 ▶).

For chemical drugs that are currently being used to treat DM, various side effects have been reported. In Iran, based on a common belief amongst patients, herbal medicine are considered alternatives to avoid side effects of chemical drug. 

*Zataria multiflora *Boiss. belonging to the Lamiaceae family*,* is an important plant which has been consumed for centuries as spice, home remedy, drug and perfume (Dauqan et al., 2017 ▶). It has been shown that this plant has antiseptic, analgesic, carminative, anthelmintic and antidiarrheal properties (Fazeli et al., 2007 ▶; Sharififar et al., 2007 ▶). In traditional medicine, antinociceptive, antimicrobial, spasmolytic, and anti-inflammatory effects have been attributed to this plant (Dadashi et al., 2016 ▶; Alavinezhad et al., 2017 ▶). Furthermore, *Z. multiflora* antioxidant properties have been proven, showing that this plant plays a role in reducing oxidative stress (Sharififar et al., 2007 ▶; Samarghandian et al., 2016 ▶).

Therefore, this plant might reduce DM complications, which if not treated, contributes to overproduction of ROS (Sajed et al., 2013 ▶). 

Our goal was not only to check the antioxidant activity, but also to evaluate the impact of this anti-oxidative product on some diabetic tissues dysfunction. The most active constituents of *Z. multiflora *are thymol, carvacrol, p-cymene, γ-terpinene, linalool apigenin, luteolin, 6-hydroxyluteolin, and β-sitosterol, which are more soluble in hydroalcoholic preparations (Sajed et al., 2013 ▶). Considering the properties of this plant, this study was conducted to evaluate the effects of hydroalcoholic extract of *Z. multiflora* on liver histopathological changes, TNF-α production, lipid profile, and the status of oxidative stress by investigating malondialdehyde (MDA), super oxide dismutase (SOD), total antioxidant capacity (TAC), and insulin level in diabetic rats.

## Materials and Methods

**Animals**

At the beginning of the experiment, 60 male Sprague-Dawley rats (weighing 200–300 g) were purchased from the Laboratory Animals Research Center (Shiraz University of Medical Sciences, Iran). The animals were acclimatized to the laboratory conditions for two weeks prior to initiation of the experiments. They were fed with rodent chow (Pars Dam Co., Tehran, Iran) and water during the study. Rats were kept in stainless steel cages in groups of 5 animals per cage in a temperature-controlled (22–25°C) environment with 12 hr light/dark cycles and 55% humidity. The protocols of the study were approved by the Institutional Animal Ethics Committee of Shiraz University of Medical Sciences (Shiraz, Iran), following NIH guidelines for care and use of animals (NIH publication No. 85-23, revised in 1996).

**Extract preparation**

*Z. multiflora* (voucher No. 1106) was collected from Fars province, Iran, and authenticated by the Department of Botany at Shiraz University, Iran. The aerial parts of *Z. multiflora* were separated, washed and air-dried. Plant tissues (300 g) were milled and extracted by percolation method performed using 1000 ml of ethanol 70% at room temperature for 72 hr. After filtration, ethanol was evaporated at 40^º^C in a rotary. After all, solvent evaporation was performed by vacuum desiccator for 24 hr, and the dried extract was stored at -20ºC (the efficiency of this method was 16.5 %)

**Diabetes Induction**

In the present study, diabetes was induced by intraperitoneal (i.p.) injection of freshly prepared streptozotocin (STZ) (60mg/kg body weight; Sigma, USA) dissolved in a 0.1mol/L citrate buffer (pH 4.5), to overnight-fasted male Sprague-Dawley rats (Masiello et al., 1998 ▶). A glucometer (Accu-Chek Active, Roche, Germany) was used to evaluate blood glucose levels. Blood glucose above 300 mg/dl were considered as criteria for diagnosis of diabetes. 

**Experimental Design**

All rats were randomly divided into 6 groups of 10. One group was considered the healthy control. The treatment period was 28 days. *Z. multiflora* extract was administered by oral gavage at dosages of 250, 500, 1000 mg/kg. Animal grouping was as follows. Group I (Control), the healthy non-diabetic control rats which received 1 mL of normal saline by oral gavage; Group II (STZ), vehicle group, the diabetic control rats received 1 mL of normal saline by oral gavage; Group III (STZ+Zataria 250), the diabetic rats received 250 mg/kg of *Z. multiflora* extract; Group IV (STZ+Zataria 500), the diabetic rats received 500 mg/kg of the extract; Group V (STZ+Zataria 1000), the diabetic rats received 1000 mg/kg of the extract; and Group VI (Zataria 1000), the normal rats received 1000 mg/kg of the extract. It is worth mentioning that since it was not possible to dilute the higher dosages (500 and 1000 mg/kg) of the extract in 1 mL of normal saline, rats of groups IV and V received 250 mg/kg of the extract 2 and 4 times more than than group III, by oral gavage.

Body weight and blood glucose levels were monitored weekly. At the end of the treatment period, rats were fasted for 12 hr and approximately 5 mL of the whole blood was collected by cardiac puncture under anesthesia. The whole blood sample was then centrifuged at 3500 rpm for 15 min and the sera were separated. Each serum sample was stored in a clean sterile micro centrifuge tube at −80ºC until further analysis.

**Determination of Biochemical Parameters**

*Liver enzymes measurement*

To evaluate the severity of liver damage, liver enzymes (ALT and AST) levels were determined using an enzymatic colorimetric method by a biochemical AutoAnalyzer device. Kits were purchased from Pars Azmoon Co, Iran. 

*Lipid profile measurement*

Lipid profile [total-cholesterol (TC), triglycerides (TG), low density Lipoprotein-C (LDL) and HDL-cholesterol (HDL-C)] was evaluated using an enzymatic colorimetric method by a biochemical AutoAnalyzer device. Kits were purchased from Pars Azmoon Co, Iran. 

*Antioxidant assay*

SOD (super oxide dismutase) and TAC (total antioxidant capacity) levels were evaluated by an enzymatic colorimetric method, using enzymatic kits purchased from Zellbio Co, German. MDA (malondialdehyde) activity was evaluated by spectrophotometric with TBARS method (Kalaivanam et al; 2006 ▶).

*Measuring plasma levels of insulin and TNF-α*

Plasma levels of insulin (RAT insulin, Mercodia, Sweden) (Meites, 1986 ▶) and TNF-α (Diaclone, France) were measured by ELISA methods (Noh et al., 2013 ▶).

*Liver histopathology*

The collected tissues were processed and sectioned at a thickness of 5 µm and stained with Hematoxylin and Eosin (H&E). The sections were then dehydrated, cleared, and eventually mounted in entellane (Merck Co., Germany); they were then cover-slipped. The prepared slides were examined under light microscopy (Olympus, Japan) at 4- 40X.

**Statistical analysis**

The data are presented as mean±standard deviation (SD). One-way analysis of variance (ANOVA) followed by *post-hoc* Tukey’s multiple range tests, were used to make comparisons among the groups. The statistical analysis was performed using SPSS (version 22.0 for windows). P-values less than 0.05 were considered to be statically significant.

## Results

**Body weight**

Diabetic groups significantly lost body weight as compared to the healthy control group (p<0.001). However, there was no significant difference in body weight between groups treated with different concentrations of *Z. multiflora* and the diabetic control group (p=0.723); as also, no significant changes in body weight was observed when comparing *Z. multiflora *1000 mg/kg treated non-diabetic group with the healthy control group (p=0.554) (Table 1).

**Blood glucose and insulin levels**

Blood glucose was significantly (p<0.001) increased, but insulin was significantly decreased in the diabetic group compared to the healthy control group. Table 1 shows a significant (p<0.001) reduction in the blood glucose in the *Z. multiflora*-treated groups compared to the diabetic group (p<0.001). Insulin level was increased in the *Z. multiflora*-treated groups as compared to the diabetic group; however, it was not statistically significant (Table 1). Also, there was a significant difference in insulin levels between the *Z. multiflora* 250 mg/kg treated group and the healthy control group (p=0.01). In Z*. multiflora*-treated groups, insulin level increased, but did not reach that of the healthy control group (p=0.143). 

**Table 1:** Nody weight, blood glucose levels, and levels of liver damage parameters (ALT, AST and total protein serum) in *Z. multiflora*-treated and control groups, at the end of study

Group	Weight (g)	Glucose (mg/dl)	Insulin (ng/ml)	ALT (IU/ml)	AST (IU/ml)	Total protein (mg/ml)
control	318.7±5.3^a^	82.3 ±2.6^a^	1.44±0.18 a	59.3±4.22 a	135.6±11.5^a^	72.1±1.0^a^
STZ	208.7±8.8^b^	483.4±18.9^b^	0.39±0.07 b	151.2±9.0^b^	296.3±20.1^b^	50.0 ±3.0^b^
STZ+Zataria250	224.6±6.3^b^	377.2±24.9^d^	0.705±0.1 b	128.8±14.4^b^^c^	277.2±7.0^b^	56.0±2.0^b^
STZ+Zataria500	227.3±7.1^b^	308.1±14.3^c^^d^	0.925±0.057 ab	105.3±16.5^c^	200.5±13. 9^c^	59.3±5.2^b^
STZ+Zataria1000	243.4±8.5^b^	258.3±27.2^c^	0.920±0.042 ab	86.0±7.0^a^^c^	186.7±18.7^a^^c^	70.0±3.0^a^^c^
Zataria1000	296.8±16.1^a^	77.2±2.28^a^	1.36±0.27 a	48.8±3.4^a^	131.2±14.1^a^	71.8±1.6^a^

STZ: Streptozotocin 60mg/kg; STZ+Zataria 250 mg/kg: streptozotocin+*Z. multiflora* 250 mg/kg; STZ+Zataria 500 mg/kg: streptozotocin+*Z. multiflora* 500mg/kg; STZ+Zataria 1000 mg/kg: streptozotocin+*Z. multiflora* 1000 mg/kg.

a, b, c, and d : According to *post-hoc* Tukey test which was used for intergroup comparisons, groups with same superscripts are not significantly different at *α*=0.05 (p≥0.05). However, dissimilar letters indicate a significant difference (p<0.05).

**Liver enzymes levels**

Plasma AST and ALT levels significantly (p<0.001) increased in the diabetic group compared to the healthy control group, but significantly (p<0.001) decreased in the *Z.* *multiflora* treated groups (i.e. Zataria 500 and 1000 mg/kg groups) compared to the diabetic group (Table1). There was a significant difference between the *Z. multiflora* 500 mg/kg treated group, and the healthy control group (p<0.001, p=0.027). Additionally, ALT and AST serum levels did not significantly change following treatment with* Z. multiflora* 1000 mg/kg non-diabetic group as compared to the healthy control group (Table 1).

**Total plasma protein levels**

It was also revealed that total plasma protein was increased in *Z. multiflora* treated groups compared with the diabetic group. A significant difference (p<0.001) was only observed between the diabetic rats treated with* Z. multiflora* 1000 mg/kg and the diabetic control group (Table 1).

**Serum lipid profile**

The results showed that there was a significant change in serum lipid profile of diabetic group compared to the healthy control group. Cholesterol, LDL and TG were significantly increased (p<0.001), but HDL decreased (p=0.001) in the diabetic group compared to the healthy control group.

**Figure 1:**
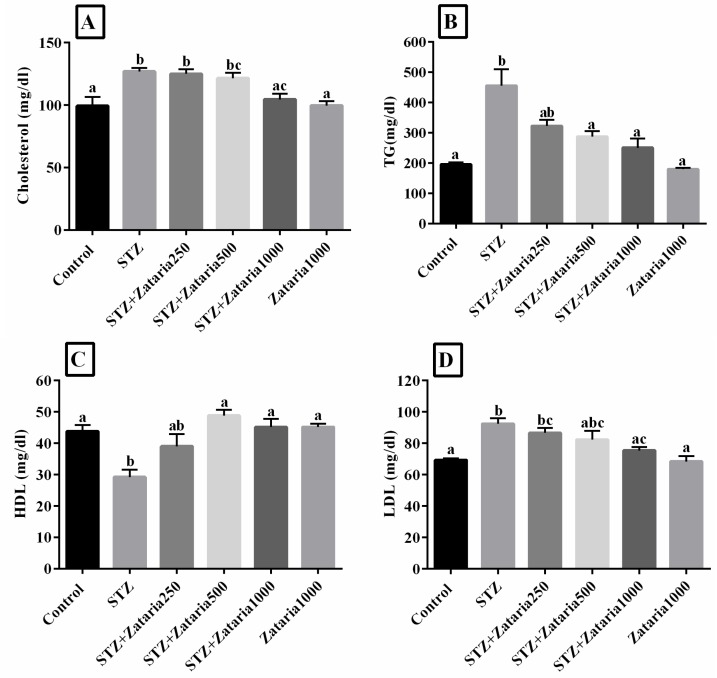
Evaluation of lipid profile in experimental groups. Figure A-D: Control: healthy control; STZ: Streptozotocin 60 mg/kg; STZ+Zataria 250 mg/kg: streptozotocin+ *Zataria* *multiflora* 250 mg/kg; STZ+Zataria500mg/kg: streptozotocin+ *Zataria* *multiflora* 500 mg/kg; STZ+Zataria 1000 mg/kg: streptozotocin+ *Zataria* *multiflora* 1000 mg/kg; *Zataria *1000 mg/kg:* Zataria* *multiflora* 1000 mg/kg

The average plasma cholesterol and LDL levels were reduced in the *Z. multiflora*-treated groups compared to the diabetic group; however, a significant difference (p<0.001) was only observed between *Z. multiflora* 1000 mg/kg diabetic group and the diabetic group (Figure 1A and D). A significant difference in plasma LDL level was observed between *Z. multiflora* 250 mg/kg-treated group and healthy control group (p=0.026) (Figure 1D). TG concentration was significantly reduced in the *Z. multiflora* 500 and 1000 mg/kg diabetic group compared to the diabetic group (p=0.021 and p=0.001, respectively). Although *Z. multiflora* 250 mg/kg-treated group showed lower levels of serum TG, the changes were not statistically significant as compared with the diabetic group (Figure 1B). According to Figure 1 C, an increment was observed in the HDL serum level in the *Z. multiflora*-treated rats in a dose-dependent manner; this increase was significant when *Z. multiflora* 500 and 1000 mg/kg groups were compared to the diabetic group (p<0.05). 

a, b, and c: According to *post-hoc* Tukey test which was used to make intergroup comparisons, groups with same superscripts were not significantly different at *α*=0.05 (p≥0.05). However, dissimilar letters indicate a significant difference (p<0.05).

**Antioxidant activity and inflammatory factors**

**Figure 2:**
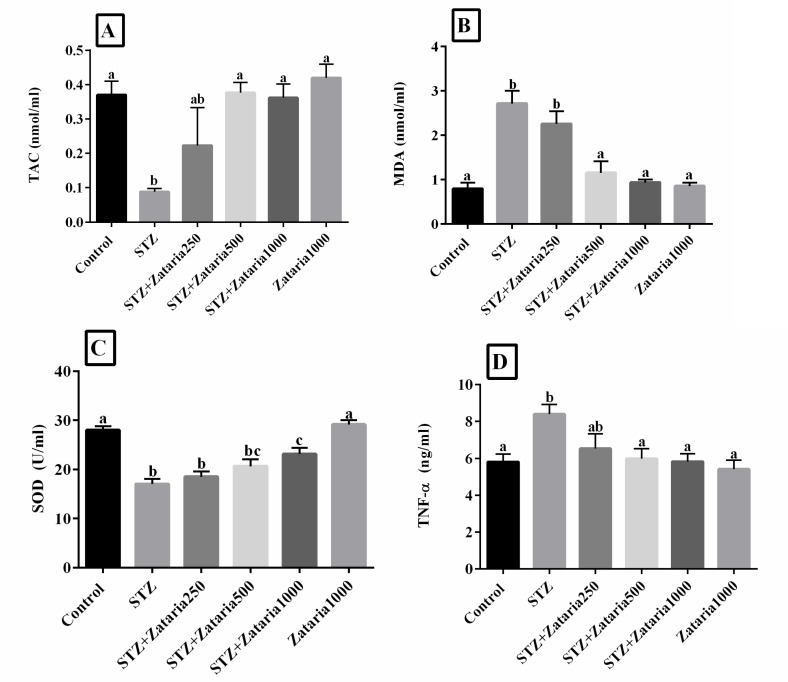
Evaluation of TAC, MDA, SOD, and TNF-α levels in experimental groups. Figure A-D: Control: healthy control; STZ: Streptozotocin 60mg/kg; STZ+Zataria 250 mg/kg: streptozotocin+ *Zataria* *multiflora* 250 mg/kg; STZ+Zataria 500 mg/kg: streptozotocin+*Zataria* *multiflora* 500 mg/kg; STZ+Zataria 1000 mg/kg: streptozotocin+ *Zataria* *multiflora* 1000 mg/kg; *Zataria *1000 mg/kg:* Zataria* *multiflora* 1000 mg/kg; TAC: Total antioxidant capacity, MDA: Malondialdehyde; SOD: Superoxide dismutase, and TNF-α: Tumor necrosis factor-α.

Data analysis showed a significant reduction in TAC (p=0.01), SOD activity (p<0.001) and total plasma protein (p<0.05) in diabetic control group compared to healthy control group. In addition, the results showed a significant increase in MDA (p<0.001) and TNF-α (p=0.01) levels in the diabetic group compared to the healthy control group (Figure 2A-D). It was also revealed that serum SOD was increased in *Z. multiflora*-treated groups compared with the diabetic group. A significant difference (p=0.007) was only observed between *Z. multiflora* 1000 mg/kg diabetic group and diabetic control group (Figure 2C). In addition, serum SOD activity in diabetic rats treated with *Z. multiflora* 1000 mg/kg was not within the range of the healthy control group, and showed a significant reduction (p<0.001). Our results showed a significant difference between the *Z. multiflora* 250 and 500 mg/kg diabetic groups and the healthy control group (p=0.004 and p=0.036, respectively), (Figure 2C). Additionally, serum MDA and TNF-α were reduced in the *Z. multiflora* 500 and 1000 mg/kg diabetic group (p<0.001 and p<0.05, respectively) compared to the diabetic group (Figure 2B and D). Serum TAC was increased in the *Z. multiflora* 500 and 1000 mg/kg diabetic groups compared to the diabetic group (p=0.023 and p=0.034, respectively) (Figure 2A). 

a, b and c: According to *post-hoc* Tukey test which was used to make intergroup comparisons, groups with same superscripts were not significantly different at *α*=0.05(p≥0.05). However, dissimilar letters indicate a significant difference (p<0.05).

**Liver histopathology**

**Figure 3:**
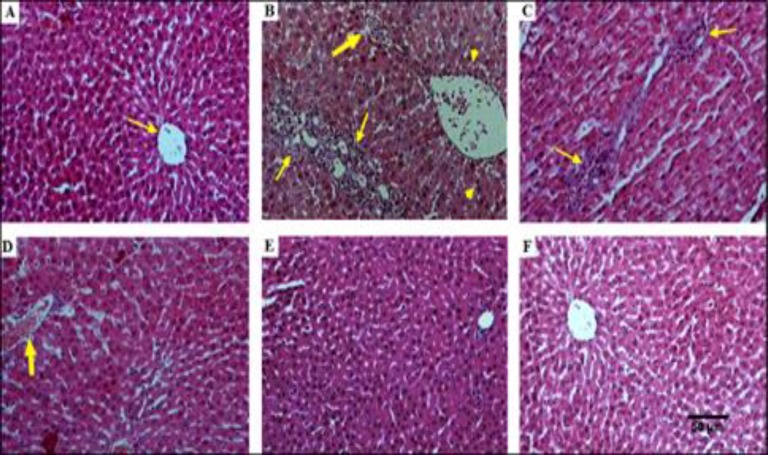
A: Histopathological study of liver tissue and effect of hydroalcholic extract of *Zataria. multiflura* in liver tissues in diabetic rats. H&E staining with magnification at X100

Liver sections of the healthy control group did not show any histological changes during the study period, and the hepatocytes were normal with normal radial arrangements around the hepatic cords (Figure 3A). In the diabetic group, the liver showed several alterations including aggregation of the lymphocytes among the hepatocytes, cloudy swelling and vacuolization of the cytoplasm, mononuclear inflammatory cells infiltration with congestion and hemorrhage, as well as hydropic changes and Kupffer cell hyperplasia (Figure 3B). The liver of the diabetic rats treated with 250 mg/kg of *Z. multiflora *extract showed no pathologic changes compared to the diabetic group. However, fewer pathological changes and ameliorated liver architecture were observed in groups treated with *Z. multiflora *extract 500 and 1000 mg/kg. Hence, high doses of *Z. multiflora* extract might reduce the severity of these changes (Figure 3D and 3E). Furthermore, in the healthy group that received *Z. multiflora* extract, no signs of pathological damages were found in the liver tissues (Figure 3F). 

A: Healthy control rats with normal hepatocytes and normal central vein (thin arrow). B: Diabetic control rats with an increment in infiltration of the lymphocytes (thin arrows), congested and edematous portal vein and hemorrhage (thick arrow). Also, vacuolation in the cytoplasm of the hepatocytes appeared as indistinct clear vacuoles (pick arrows). C: Diabetic rats treated with *Z. multiflora* 250mg/kg showed lymphocytic inflammation and vacuolization of cytoplasm (thin arrow). D: Diabetic rats treated with *Z. multiflora* 500 mg/kg showed fewer pathological changes and improved liver architecture. E: Diabetic rats treated with *Z. multiflora* 1000 mg/kg with relative restoration of the liver architecture and normal hepatocytes. F: Non-diabetic rats treated with *Z. multiflora* 1000 mg/kg with normal liver histopathological features.

## Discussion

Diabetes mellitus as a complex metabolic disorder, has imposed a huge burden on the society and has threatened the health of millions around the globe. Various chemical drugs are used to treat DM; however, they possess various side effects. Therefore, safer modalities with fewer side effects are required. Nowadays, herbal therapy has gained much attention and it is believed that plants and their derivatives can ameliorate the complications of diabetes, and they are regarded as safer treatments compared to the chemical drugs. In the present study, the effect of hydroalcoholic extract of *Zataria multiflora *Boiss. was evaluated in a diabetic rat model. In this study, it was revealed that the *Z. multiflora *extract was able to reduce hyperglycemia, liver enzymes abnormality, inflammation and oxidative stress in comparison to the diabetic control group. In addition, based on the biochemical and histopathological results, no complications nor damages to the liver tissue was observed following treatment with 1000 mg/kg of *Z. multiflora *extract*. *Hosseinzadeh et al. (2000) ▶ showed that the maximum non-fatal dosages of *Z. multiflora *Boiss. extracts in mice and rats were 2.2 and 2 g/kg, respectively (Hosseinzadeh et al., 2000 ▶). 

In line with our findings, Samarghandian et al. (2016) ▶ showed that *Z. multiflora* extract ameliorates oxidative stress, lipid abnormality and blood glucose in rats (Samarghandian et al., 2016 ▶). Furthermore, Khoshvaghti et al., ▶ reported that *Z. multiflora* extract has a beneficial effect on plasma lipids in rats (Khoshvaghti et al., 2012 ▶). Also, Gholamhoseinian Najar et al. (2015) ▶ showed that *Z. multiflora* extract improves glucose uptake by peripheral tissues and reduces blood glucose level (Gholamhoseinian Najar et al., 2015 ▶).

Also, it was shown that flavonoids can lead to reduction in the glucose plasma (Sakai et al., 2001 ▶; Arabbi et al., 2004 ▶). This might be due to the presence of phenols, which could reduce plasma glucose levels by reducing oxidative stress (Samarghandian et al., 2016 ▶; Sabu et al., 2002 ▶). 

Antioxidant compounds can ameliorate diabetic complications. *Z. moltiflora *as an antioxidant herb improves insulin secretion and reduces plasma glucose level. Also, *Z. moltiflora *has positive effects against the detrimental effects of ROS on the pancreatic beta cells (Kavoosi, 2011 ▶). 

In another study, flavonoids decreased glucose absorption followed by the inhibition of α-glucosidases and α- amylase. Furthermore, flavonoids improve insulin secretion and glucose uptake. Hence, flavonoids play a key role in amelioration of hyperglycemia (Ghorbani, 2017 ▶).

These findings are in accordance with our results. The groups that were treated with 500 and 1000 mg/kg of the extract might partially regenerated the pancreatic beta cells, but a significant difference was not observed between these groups and diabetic control group.

The present study showed that plasma AST and ALT activity had significantly increased in the diabetic group compared to the healthy control group. The activity of AST and ALT was significantly reduced in the *Z.* *multiflora*-treated groups compared to the diabetic group, except for the *Z. multiflora* 250 mg/kg diabetic group. Elevated liver enzymes perhaps suggest that diabetes mellitus could result in gradual liver destruction. *Z. multiflora* extract contains thymol and carvacrol, which act as antioxidants and could prevent liver damage by inhibiting lipid membrane peroxidation and increasing the antioxidant enzymes (Shittu et al., 2013 ▶; Lee and Shibamoto, 2002 ▶).

The present study showed that *Z. multiflora* extract had anti-inflammatory effects on diabetic rats and could reduce plasma TNF-α level. Hosseinzadeh et al. (2000) ▶ showed that *Z. multiflora* extract had anti-inflammatory effects on acute and chronic inflammation (Hosseinzadeh et al., 2000 ▶). In another study, it was shown that *Z.* *multiflora* extract and its constituent carvacrol had preventive effects on lung inflammation and oxidative stress in animal models of COPD (Chronic Obstructive Pulmonary Disease) (Shokrzadeh et al., 2015 ▶). Terpenes such as carvacerol and thymol as well as other compounds, such as apigenin, luteolin and 6-hydroxyluteolin are bioactive components of this extract (Boskabady and Gholami Mahtaj, 2014 ▶). Additionally, it was shown that *Z. multiflora* might lead to suppression of cyclooxygenase enzymes. Hence, this plant inhibits the synthesis of inflammatory cytokines (Hosseinzadeh et al., 2000 ▶). COX is an important regulator for the conversion of arachidonic acid into inflammatory prostaglandins and eicosanoids that mediate inflammation, immunomodulation, apoptosis, and blood flow (Verma et al., 2016 ▶). Another investigation showed that *Z. multiflora* extract has anti-inflammatory components such as luteolin (Duvnjak et al., 2007 ▶). This extract had a positive effect on inflammatory bowel disease and improved inflammatory conditions (Noh et al., 2013 ▶). TNF-α is an inflammatory cytokine that is secreted by macrophages. This cytokine suppresses insulin excretion in DM (Swaroop et al., 2012 ▶). Increased level of liver TNF-α leads to the activation of NF-Kb. NF-Kb stimulates INOS (inducible nitric oxide synthase) expression that causes nitric oxide overproduction (Frances et al., 2013). Hence, these changes result in oxidative stress, which could be inhibited by *Z. multiflora* elements. 

We showed that STZ significantly leads to reduction of SOD activity and TAC and increase in TNF-α, and MDA compared with healthy control group. Our results showed a decrease in serum MDA, but an increase in SOD in the *Z. multiflora* (high doses)-treated groups compared with the diabetic group. Mohammadi et al. (2014) showed similar results for 1000 mg/kg *Z. multiflora* extract. They showed that this extract induces body weight loss, and reduces fasting blood glucose, TG, and fasting insulin, while increases adiponectin (Mohammadi et al., 2014). ROS are oxidants that damage various tissues and are associated with many diseases, such as cancer, inflammatory diseases and DM. It has been shown that phytochemicals reduce ROS and improve antioxidant system (Shittu et al., 2013 ▶). Samarghandian et al. (2016) ▶ indicated that antioxidant compounds had anti-diabetic effects (Samarghandian et al., 2016 ▶).

Finally, the results of our study revealed that body weight decreased after diabetes induction. However, in the *Z. multiflora*-treated groups, there was no significant increase in the body weight. We had hypothesized that *Z. multiflora* could prevent weight loss in the *Z. multiflora* treated groups by controlling blood glucose and reducing glycosuria during treatment.

*Z. multiflora* extract decreased oxidative stress damages and lipid abnormality. Additionally, it was able to improve the inflammatory response, blood glucose metabolism, and liver function in a dose-dependent manner, in rats with DM. 
